# A Comprehensive Review on Types, Methods and Different Regions Related to Water–Energy–Food Nexus

**DOI:** 10.3390/ijerph18168276

**Published:** 2021-08-04

**Authors:** Zeyang Bian, Dan Liu

**Affiliations:** 1 Business School, University of Shanghai for Science and Technology, Shanghai 200093, China; 2 School of Economics, Fudan University, Shanghai 200433, China; liudan2018fd@163.com

**Keywords:** water, food, energy, climate, modeling, sustainability, development

## Abstract

Water, food, and energy are three of the most important resources for long-term survival and development. The term “nexus” is used to underline the need of controlling these primary components collectively rather than separately because they are interconnected and linked. With the purpose of better understanding nexus thinking and showcasing nexus analysis approaches and tools, this study explores the current state of the approach to the water–energy–food relationship, which has gotten a lot of attention in recent years. Water–energy, water–food, water–energy–food, water–energy, and climate are the four forms of nexus. This paper examines a variety of methodologies based on their principal objectives and provides a basic overview of a wide range of currently available methods and instruments for analyzing the water–energy–food (WEF) nexus. According to this study, the quantity of studies on the water–energy–food nexus has increased significantly, as the scientific community’s ability to analyze water, food, and energy interlinkages at a greater resolution. The integration and optimization of this multi-centric nexus is explored, with focus on four regions—Asia, Europe, America, and Africa—as a case study. The WEF nexus should be used in case studies to help illustrate its intricacies. Furthermore, this study builds a methodology and frameworks to find study linkages between water, energy, food, and other components, for a nexus analysis and discuss the major challenges and its solutions. This study also includes a scientometric analysis that looks at the countries and keyword mapping. Furthermore, the study is being planned, with an emphasis on quantitative analysis of the water–energy–food nexus which is helpful for the water security at local and global scale. This study aids in the coordination of research efforts to solve the difficult issues in nexus research and create sustainable and adaptable water, energy, and food systems.

## 1. Introduction

The lack of water and the growing demand for local supply are currently and will be a challenge in the future. Like climate change, the amount and range of pollution sources continues to increase, creating increasingly unpredictable weather patterns that are increasing the chances for droughts, floods and naturally occurring catastrophes. Water and energy savings have become one of global sustainable development’s most important principles. They are also connected and interdependent. Reducing the amount of water required to produce energy can be accomplished in two ways: conserving water or diverting it to another use [1]. With a growing global concern for water and energy security, the substantial contribution pertaining to the energy–water linkages has caught the attention of the scientific community recently.

**Figure 1:**
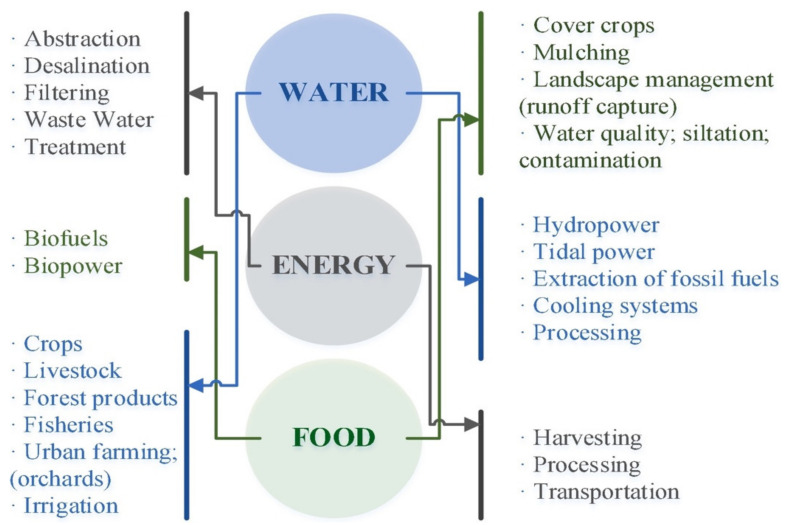
The nexus system defines the major inflows within and between water, energy, and food sectors [20].

**Figure 2:**
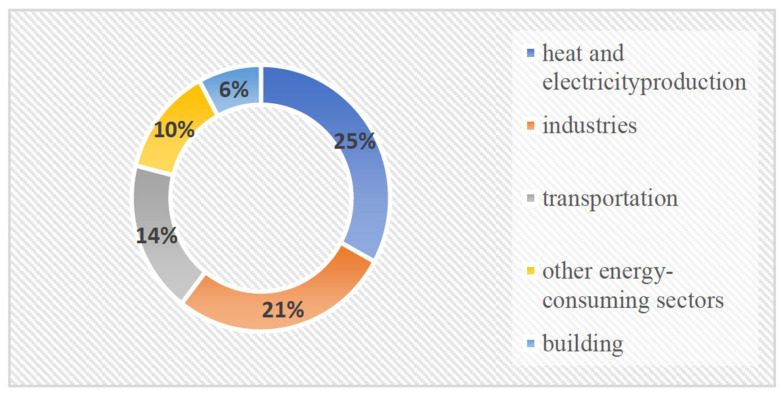
Major contributors of greenhouse gas [12].

In water–energy nexus analysis, advanced water and energy planning, as well as a better understanding of possible future policies and technologies, are coupled with knowledge of better educated water and energy planning. This is expected to assist public policymakers and energy and water resource managers in conservation and sustainability [2]. A strain is placed on three essential resources when the population increases: water, energy, and food (WEF). The United Nations predicts that the global population will reach 10 billion by the year 2100, with a majority of this increase taking place in urban areas [3]. As of 2030, 60% of the world’s population will reside in urban areas. Due to the growth in the population, urbanization, and industrialization, the demand for water is increasing and also causes stress on water supply and quality, particularly in regions where water stress already exists, such as the Asia, Central America, and Middle East North Africa (MENA) regions [4]. Climate change has an influence on water–energy–food resource availability by affecting agriculture output, water supply, and cooling and heating demand [5]. Climate change will certainly decrease rainfall, limiting access to surface water and further diminishing stored soil moisture in subtropical areas, which already have the fastest population increase and the biggest demand on water–energy–food resources [6]. A global warming scenario is likely to intensify competition and trade-offs in the three resources (food, water, and energy) that are under increasing demand [7,8]. Water and energy consumption will rise across all industries as a result of the global temperature rise caused by climate change [9]. Figure 1 illustrates the major inflows within and between water, energy, and food systems. These aspects should preferably be grouped using standard categories to facilitate the compilation of quantitative records that are consistent, rational, and comparable across time and nations. Furthermore, the more frequent and severe weather events cause a greater the threat to agriculture. Furthermore, there is evidence that climate change is having an impact on the energy industry by driving the adoption of renewable energy supply alternatives that reduce greenhouse gas emissions, leading to an increase in the use of biofuels [10]. Major contributors to greenhouse gas are shown in Figure 2: Industries and electricity production are the main sources of greenhouse gas, contributing 21% and 25%, respectively and transportation is the third source of pollution, contributing 14%. Meanwhile, buildings and other sources contribute 16% to hazardous emissions. However, climate change adaptation calls for more efficient energy, water, and food usage to combat these risks.

There are many methods for nexus research which depends on the scales, goals, and data availability. Despite this, mathematical methods may be utilized to show nexus issues of almost any scale. As a result of this, the agent-based model is more suited to reveal the consequences of people’s choices, whereas life-cycle analysis is useful in evaluating policy and technical changes linked to resource use and the environment. The integrated index also helps policymakers consider many social and environmental factors [11]. Modeling methods are not convincingly employed by policymakers due to a dearth of relation between scientists, engineers and practitioners in the energy, water, and agricultural sectors. This issue is solved by an interdisciplinary approach and by increasing the models which are more sector specific. Globalization is an issue for WEF nexus research. Trade liberalization has complicated the links between water, energy, and food since resources and commodities are constantly flowing across international boundaries. Apart from the data and integration challenges inherent in the WEF nexus, this paradigm may be studied on a variety of geographical and temporal scales [12]. Moreover, according to the study, water, energy, and food supply changes impacted water resources more in Central Asia than in South Asia. The research found that water abstraction, desalination, and treatment activities were heavily dependent on electricity throughout the Middle East and North Africa [13].

Rain-fed agriculture generates a major proportion of our food that is highly prone to reduced precipitation and has major repercussions: water is not as readily available for food and energy production when rainfall decreases [14]. Simultaneously, storm intensity and the duration between rain events are predicted to rise, impacting soil quality and agricultural productivity [15]. Longer dry periods and less precipitation will result in higher water demands, as well as worse water quality and availability. Individual storms of higher intensity, on the other hand, will almost certainly result in additional property damage, as well as greater pest and sickness exposure [16]. The ever-growing interconnections of resources, resulting from rapid levels of consumption, the problems with the supply chain and the failure of sector-oriented management techniques [17] are strengthening an integrated water–energy–food strategy. The methodology of the water–energy–food nexus is stochastic, so that random occurrences may have non-random results. Adequate resources are required for the proper functioning of the three systems as well as regulations for the safeguarding of resources. The water–energy–food nexus strategy helps communities to use objective data to develop logic, efficiency and fair methods of management of resources and socio-economic systems [18].

**Figure 3:**
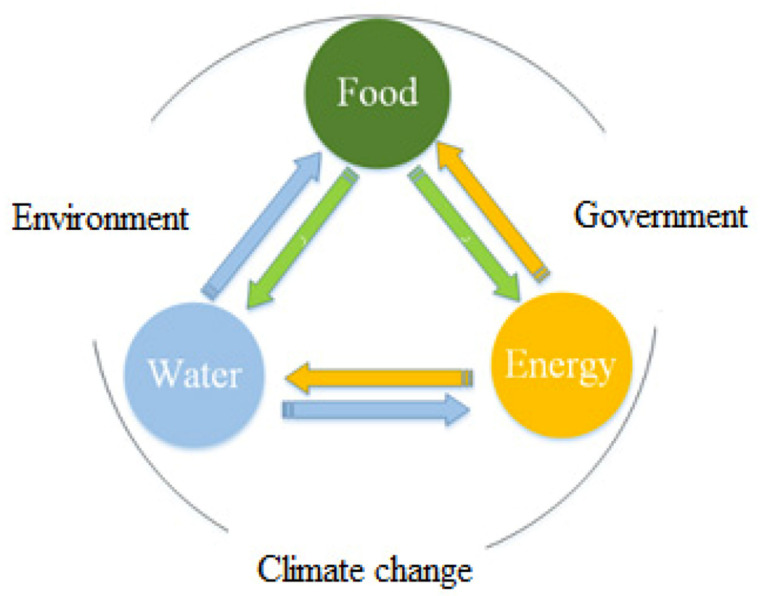
Conceptual framework of water–energy–food nexus [12].

The water–energy–food nexus governance model incorporates extreme weather events, poorly planned cities, large-scale terrorist assaults, geopolitical failure, climate change and infrastructural issues. These risks have an impact on the management of water, energy, and food resources, posing not only local but also global dangers, demonstrating the interdependence of hazards, resource management, and the world situational uncertainties. The framework of water–energy–food nexus is shown in Figure 3 [19]. Any quantification investigation to detect water–energy–food connections starts with a characterization stage, in which links between the parts of the research, as well as their quantitative values (inflows) or intensities, are identified. In addition, the context of these linkages is established. Nonetheless, the environmental, social, economic, and institutional factors that influence water–energy–food management techniques and regulations vary greatly across nations.

## 2. Significance and Outline of Current Review

The primary goal of this research is to provide an overview of modeling techniques used in current water–energy–food nexus research. Additional ‘nexus’ characteristics could be incorporated into current water and energy models, leading to model advancements. As a result, researchers and future consumers can better understand their strengths, weaknesses, and applications. The linkages between water, energy and food, their impacts on policy making climate change and socioeconomic development are discussed in this paper, which is beneficial to achieve the objective of nexus and achieving the sustainable expansion.

By examining and categorizing current water–energy–food nexus case studies and evaluation methodologies, this article proposed a framework to provide a systemic solution to the receding issue. Therefore, this review aims to contribute to the development of water–energy–food nexus approaches that add value to the field while avoiding duplication of effort. This review has three specific goals: (1) to gain a better understanding of the ongoing case progress; (2) to present current water–energy–food nexus tools and assess their suitability, scope, and degree of necessity in a variety of nexus settings; and finally, (3) to bring the discussion to a close, we should consider the future development of techniques for water–energy–food nexus analysis.

**Figure 4:**
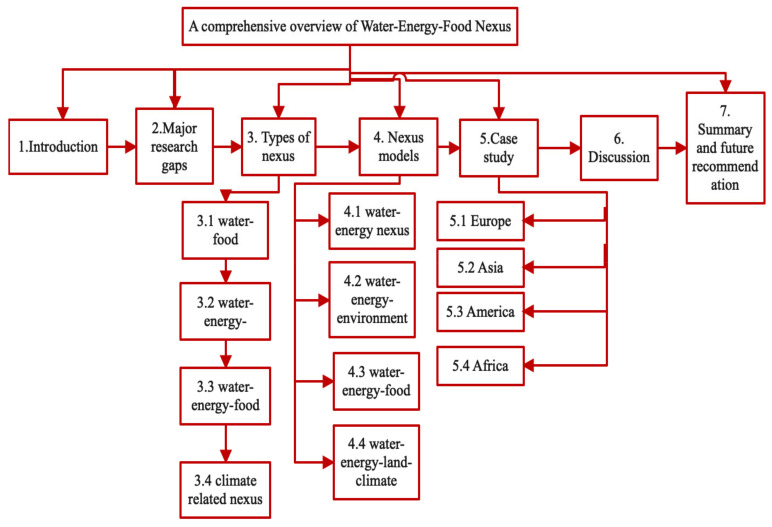
Outline of the study.

Future study on regional resource management might be guided by these findings. Additionally, this article discusses chosen case studies from each continent—Asia, Africa, Europe and America—in order to ensure that each location is represented, hence strengthening the study’s uniformity and effect. Further scientometric analysis is also performed in this review. The major difficulties for imposing this idea are classified as a lack of unified policy and measures for the network, data insecurity and a high number of subsystem data, system boundaries, a lack of standards and regulations, and a lack of efficient software platforms. The outline of the study is shown in Figure 4.

## 3. Types of Nexuses

**Figure 5:**
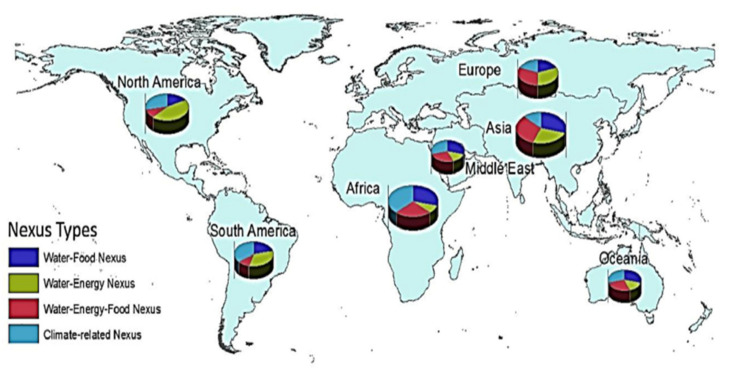
A graphical representation of nexus types [27].

**Figure 6:**
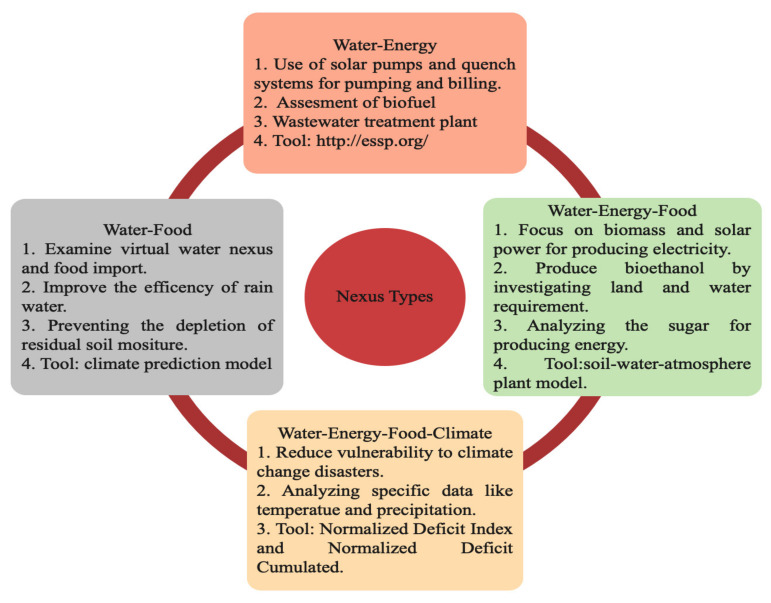
Nexus types [27].

Four types of nexuses are shown in Figure 5 and Figure 6 shows the graphical representation of water–energy–food nexus. America is more concerned about water–energy nexus while Europe and Asia deal with water-energy–food nexus, further Africa is more concerned with climate related nexus. The Middle East and Oceania share equal portions in all nexus types.

### 3.1. Water–Food Nexus

To address the water–food nexus actions were taken to reduce water use and increase water efficiency in order to produce food more effectively. The environmental impact of food imports and the virtual water nexus was studied [21], different techniques were used for decreasing the consumption of water by shifting to low water consuming crops and enhancing the efficiency of green water usage by shortening the fallow time and avoiding following crop harvest in order to prevent the remaining soil moisture in the field [22]. Through extension and training programs, stakeholder-led [23], microfinance model, public-private partnership and power pricing systems in the agriculture sector were observed. To incorporate other programs and operations, data-intensive methods like climate prediction for agriculture were developed.

### 3.2. Water–Energy Nexus

Projects using water and energy ranged from water for energy to energy for water. Hydropower production and water-based biofuels are two examples of harnessing water to generate energy. Pumping water for food and purifying wastewater with electricity are two instances of energy usage. Agricultural irrigation, for example, has seen a significant increase in its energy demand in the Spanish water industry [24].

Environmental initiatives included a discussion of bioenergy sources like as microalgae, water reservoirs obtained from abandoned mines, and solar pumps and billing quench systems [25]. Simultaneous implementation of tariff and investment policies, as well as investigation into water treatment plant efficiency with respect to shale gas development, promoting well-regulated on-site treatment technology and examining carbon and water pricing scenarios were all identified as essential market management techniques [26]. The project teams implemented several social and governance initiatives to help them accomplish quantitative fine-scale site-specific data for quantifying stakeholder participation in the water–energy nexus. Website content sharing is another way to promote the energy–water nexus.

### 3.3. Water–Energy–Food Nexus

Integrated water resource management enhanced the activities of the water–energy–food nexus. Other biofuel-related initiatives included focusing on Mauritius, South Africa, and China, analyze sugar in order to produce alternative energy, concentrated solar power for creating electricity, and using woody biomass to produce electricity in the African country of South Africa.

Concerning groundwater, a study in Ethiopia and Iran revealed that reducing irrigation application might reduce energy usage and carbon emission. A variety of integrative regional management models were investigated in Central Asia that focused on promoting the expansion of the market for electricity, investing in hydropower projects, reforming the irrigation sector, and encouraging the development of regional public goods.

### 3.4. Climate Related Nexus

A large number of climate-related actions have been carried out in order to lessen long-term susceptibility to catastrophe and environmental degradation caused by climate change. Several climate-related nexus projects employed a variety of approaches, such as examining specific data, including the precipitation and temperature in Mexico, utilizing meteorological and historical data to underpin the climate change and poverty nexus in Nigeria, and emphasizing energy use and greenhouse gas emissions in water management. Additionally, climate change nexus was also investigated in cities. To resolve a major water scarcity in the Australian National Electricity Market in 2007, a system dynamics technique was employed. Resilience and sustainability were improved through the use of hybrid energy solutions such as the transformation of municipal governance systems [27].

## 4. Major Gaps in Nexus Research

The World Economic Forum’s nexus notion has been criticized. The main points of contention include the nexus studies; there seems to be a lack of concentration, the assertion that the method is not new, particular sectors lack of integration (e.g., ecosystems), and the absence of shared techniques for analyzing nexus issues. Regarding the concept, many researchers argue that the nexus is still an expanding concept [28], is relatively immature [29], and is narrative, but not useful in applications [30], and is without any common definitions, methods, and frameworks [31,32,33,34]. Furthermore, the implementation of the nexus idea has gotten a lot of attention from academics. For example, Mitchell et al. [35] and Wichelns [29] cautioned that using the nexus method and incorporating a large number of stakeholders in policy-making processes, particularly in developing nations, may result in delays, sluggishness and inertia. However, stakeholders’ engagement is thought necessary for accurate nexus mapping and comprehension. Additionally, current nexus implementations have been unable to handle complicated interconnections owing to a lack of boundary specification [36] and lack of data sharing and availability [37]. There have been criticisms leveled at the intended effect of implementing the nexus idea in many locations across the globe. Among the critiques is a failure to take into account inherent political issues [38], the primary democratic aim of long-term viability [34], gender issues, as well as national-level integration of programs, policies, and institutions [32] as well as the implementation of the World Economic Forum Nexus in the decision-making process [39]. Table 1 shows the challenges and solutions as related to water–energy–food nexus.

## 5. Different Modeling Techniques

In the past, many modeling tools for water and energy system analysis were presented. Based on several factors, these approaches are divided into two categories. Water and energy are intertwined in the water–energy nexus. The water–energy environment nexus, or the linkages between water and energy while taking environmental variables like climate change into account, is the second category. The third category, the water–energy–food nexus has gotten a lot of attention in recent years since it demonstrates the connection between water, food, and energy. The water–energy–land–climate nexus techniques category includes nexus tools that take climate change’s effects on water, energy, and land usage into account. Real-world issues are simplified using modeling approaches. This review study is useful in outlining the most viable hydro scheduling modeling and solution methodologies.

### 5.1. Water–Energy Nexus

#### 5.1.1. Energy Intensity

EI (energy intensity) is a hybrid top-down/bottom-up model designed to assess energy fluxes in urban water systems. The goal of the top-down model is to produce high-level monthly energy intensity estimates for the whole metropolitan water system. The bottom-up model was created to determine precise energy estimates for a portion of the city’s water supply region. In the case study of the Northern California, it is an accounting approach [40]. However, there was no scenario function and no program that could be used with this manner.

#### 5.1.2. Linkage Analysis

The use of resources, both direct and indirect, as well as the participation of each economic sector, may be detected using linkage analysis, which is developed from input-output analysis (resource providers or resource consumers) [41]. Linkage analysis was utilized to investigate the virtual water and embodied energy between urban economic sectors in Beijing [42]. The resources effects from economic activity are divided into four categories: an internal effect (IE), a mixed effect (ME), and backward or forward net linkages (NBL and FNL) (NFL). Beijing was offered as an example of all the variables, including equations and ramifications [42]. It is a quantitative way for analyzing the water–energy nexus (WEN) from an economic standpoint.

#### 5.1.3. Multi-Regional Nexus Network

The MRNN (multi-regional nexus network) was created using input-output models, like input-output (MRIO) multiregional and the ecological network analysis (ENA) model, to comprehensively quantify the city- and regional-level water and energy supplies [43]. The MRIO model is used to calculate water and energy fluxes that are not straight, apart from the direct flows, and to calculate the quantity of water and energy necessary to produce commodities and services in a complex system [44]. Whereas MRIO measures the total financial flows to and from the transformation process, ENA utilizes the integral flows to assess both the direct and indirect financial flows from and to the transformation process as well as the interconnections between the various economic sectors [45,46]. Additionally, ENA can track energy or water consumption processes backwards in time to capture the whole consumption or use as well as reveal the system’s structure and function [47]. This method has been employed in the context of urban agglomeration in case studies of the Beijing-Tianjin-Hebei region [43]. The qualitative and quantitative nexus methods are equally significant to a quantitative nexus understanding.

#### 5.1.4. Urban Water Optioneering Tool

The urban water optioneering tool is a water instrument that specifically concentrates on urban water systems [48,49,50]. This instrument was created by the National Technical University of Athens, unlike the EI approach [48]. In addition, the water-related images have been included in the water tool box. It has two noteworthy benefits: estimation of energy consumed by water appliances, and evaluation of effects of green spaces on urban heat island effect.

This previously helped the city of Athens to create and quantify a wastewater reuse plan for the water system [51]. Baki and Makropoulos [52] conducted research into the influence of energy consumption on urban water supply systems, which led to an extension of the University of Washington Tool for Urban Water Utilization Analysis (UWOT), available for strategic planning and management of water and energy resources in cities that aim to be environmentally friendly. This software does not have a scenario feature.

#### 5.1.5. Great Lakes Energy Water Model

GLEW (Great Lakes Energy Water Model) is an integrated system dynamic model intended to simulate electricity generation and associated water needs in the Great Lakes Region, as well as environmental health [53,54]. This machine may be controlled using the Studio Expert 2008 software platform created by Powersim. More than half of the water withdrawn in the Great Lakes Region is used for thermoelectric power generation. A significant hazard to water flows and environmental health may arise due to the significant water use for electric generation. This method was used to investigate the repercussions of alternative energy portfolio characteristics on the water resources of this Basin [54].

### 5.2. Water–Energ–Environment Nexus

#### 5.2.1. ZeroNet DSS

The ZeroNet DSS (ZeroNet Decision Supporting System) is a unified model architecture that enables water owners and managers to synthesize critical water supply and demand data and prepare for shortages [55]. It consists of three primary components: watershed tools, a rapid scenario tool, and a knowledge base, all of which are built on the foundations of watershed data, stakeholder input, and GIS capabilities. Users may easily evaluate data and their own situations using this technique, which features a decision support system user interface. This system was created for the San Juan River Basin’s water management, and it is exclusively applicable to energy water consumption.

#### 5.2.2. WATER

The Argonne National Laboratory’s WATER (Water Analysis Tool for Energy Resources) is a web-based application that assesses the water footprint of the various phases of bioenergy production, from feedstock production through conversion to bioenergy, with geographical resolution at the national, state, and regional levels. It also aids in the evaluation of the effects of water use on water quality. This model offers a temporal resolution from day to year, as well as a geographic resolution from regional to national scales. This application enables potential users (including government, American businesses, researchers and the general public) to compare pathways, generate scenarios, and analyze regional feedstocks to aid in the growth and planning of the bioenergy sector [56,57]. It is useful, but it must be utilized for the assessment of U.S. bioenergy production and transportation and hence needs extensive data input, which includes a detailed study of climate, water resources, land use and water data processes.

#### 5.2.3. Computable General Equilibrium

A recursive multi-sectoral dynamic (CGE) model is employed to investigate how an increase in energy taxation effects water extraction and usage of energy in China [58]. The CGE model is built using a generalized algebraic modeling framework that incorporates production modules, pricing, income spending, an equilibrium, trade and a macroeconomic completion [59]. The pricing module includes tax, which is included into the CGE model, as one of the three primary inputs. Other components include the number of sectors and the price of the energy goods. The CGE model can explain interrelationships across economic sectors, including interdependencies between them, and can show how a multiplicity of economic factors interrelate with policy instruments [60]. Simulating the energy taxation consequences on water extraction and energy usage in China involved integrating many models into one [58].

### 5.3. Water–Food–Energy Nexus

#### Transboundary River Basin

The TRBNA (Transboundary River Basin Nexus Approach) is an integrated model that uses trade-offs and effects to identif, and propose feasible national and transboundary policy measures and technical initiatives to minimize intersectoral conflicts [61]. The purpose of regulating the nexus is to be supported. As a series of nexus evaluations, it has been used to analyze selected river basins under the United Nations Economic Commission for Europe’s Water Convention [62]. This model structure is made from six steps: socioeconomic and geographic factors evaluation, recognition of important sectors and players, sector perusal, cross-sector concerns evaluation, discourse around relevant topics, and determining benefits. However, the only relationship from implementation to solutions that are implemented is by way of a nexus in the final phase.

### 5.4. Water–Energy–Land–Climate Nexus

#### Platform for Regional Integrated Modeling and Analysis

PRIMA (Platform for Regional Integrated Modeling and Analysis) is a PNNL-developed integrated model that simulates interactions that are complicated between climate, energy, water, and land at decision-relevant geographical scales. The climatic model, socio-economic model, hydrological model, agricultural model, building model, electrical model, and others are all combined into one system. At regional levels, plans to address complex socioeconomic and environmental change are developed and evaluated with the help of stakeholders [63,64]. PRIMA may also be used to investigate the effects of regional climate change and sea level rise on energy infrastructure throughout the Gulf Coast of the United States. Velo is a PNNL-developed open-source software infrastructure for orchestrating processes and managing projects, material, and metadata. It also enables users to do simulations [65,66]. This software’s design is adaptable, allowing it to bring the component models together depending on the situation, in a number of ways. However, because of the high data needs, this method is only employed in a few areas of the United States [64].

## 6. Case Study from Literature

The WEF nexus notion is often considered ambiguous in the literature, and there is no agreement on how it should be defined. Thus, illustrating the WEF nexus via case studies should aid in elucidating its complexity [57].

### 6.1. Case Study of Europe

A unique challenge in Europe for the WEF nexus is that of balancing energy prices for farmers while allowing them to afford irrigation expenses. Distributing water to smaller communities is also a primary concern, especially for hydropower, and increasing the public’s awareness of the environment is necessary as well [67]. In the administration of the World Economic Forum, uncertainty is a recurring theme. As a result, European nations must adopt ideas and strategies for crisis prevention to boost strength and avoid difficulties associated with the World Economic Forum’s security. The nexus concept of the World Economic Forum illustrates complex relationships between stakeholders and sectors. Few nations, however, have policies and strategies in place to address the WEF nexus as a whole. For example, the United Kingdom lacks policies that mark the WEF nexus interconnection, promoting trade-offs across WEF sectors and weakening the country’s ability to withstand climate change [16]. Despite recent advances in the European Union in acknowledging nexus issues, most that provide consistent source strategies remain sector-specific, confronting medium- and long-term concerns about resource management and climate change adaptation. Dynamism, adaptability, multidisciplinary, and science must govern new policies and decision-making. According to research conducted with UK experts, assessment and analysis in the context of the WEF nexus need a multi-stakeholder and transdisciplinary approach across the knowledge creation and decision-making processes. This strategy necessitates active participation of stakeholders from all sectors [68].

Increased worldwide demand for natural resources, along with climate change, has created substantial issues in urban development and sustainability in Europe. Munich, for example, one of Germany’s major cities, is heavily reliant on food imports. In this regard, [69] green agriculture might use vertical farming as a way to increase sustainable food production. Vertical crops may provide 66% of Munich’s fruit and vegetable needs and almost half of the city’s vegetable needs. Another benefit of vertical farming is that it reduces heat islands, which helps to reduce energy consumption and keep buildings cool in the summer. Similarly, wastewater treatment and recycling might lead to water savings of up to 26% when combined with rainwater collection. Since the water saved may be utilized for vertical farming in urban agriculture, this project’s water use has been conserved. It can use the integrated WEF nexus methodology to improve the city’s sustainability, with the addition of WEF security by bringing together the greatest diversity of WEF resources stakeholders, a sophisticated and broad system of communication allows participants to reduce interdependent risks.

### 6.2. Case Study of Asia

It is vital that the worldwide population be fed since the Asia-Pacific area takes up two-thirds of the global food supply and uses about 60% of the world’s fresh water. Due to the fact that South Asia has just 3% of the world’s usable land and about one-fourth of the world’s population, with 1.6 billion people, just around 1.6% of the world’s landmass is in South Asia and one-fourth of the world’s population is in the South Asian region [7]. Given the several forces influencing the area, including a high demand for food production from a restricted quantity of land, limited water supplies, and higher energy costs, several complicated scenarios exist. Natural resources are becoming more scarce as the population is on the rise in the area, and this has the potential to further affect the environment and contribute to global warming [7,62]. According to [7], there are distinct obstacles and complications in the Himalaya region of Asia. A high rapid population growth, poverty, paucity of resources, and a shortage of land characterize this area. Water and energy used in food production, in combination with greenhouse gas emissions and deforestation, poses a danger to ecosystems, disrupts development, and raises hazards in regions all over the world. To tackle the WEF nexus issues and promote sustainable development, we must use a transboundary strategy. From a comprehensive perspective, it is feasible to devise strategies that mitigate the impact of hazards or prevent them from developing. To prevent the negative impacts of unilateral resource exploitation, nations must work together to control the natural resources they have in common. In particular, rivers and other shared resources often span borders, which necessitates cooperation in order to avoid unilateral exploitation [70]. One country’s unilateral exploitation of a river produces instability for other nations, necessitating management under the WEF nexus model to achieve a better regional security balance. This case study shows that an increase in hydroelectric power plant installations in the area undermines food security. To do this, governments from various nations may have to implement public policies that address resource competition, namely those linked with the WEF nexus [71]. In hopes of addressing the future water supply for electricity and food production in the basin, Yang et al., developed a hydro-economic model for the Brahmaputra River Basin. The findings show that societal and environmental catastrophes might be avoided if the region’s main river basins get adequate precipitation. However, the patterns of precipitation and temperature are predicted to be affected by climate change, exposing the area to threats and disasters. Due to this uncertainty, which may apply to most of Asia, the WEF nexus strategy plays a critical role [71]. Additionally, depletion of river water in other nations might make it difficult for one or more of those nations to provide water for both food and energy production, with a disproportionate impact on the poor, in particularly those living in riparian zones. Stakeholders should build a cross-border strategy to solidify a sustainable solution to the nexus problems in the area.

### 6.3. Case Study of Africa

The WEF nexus is examined in different settings. It views the role of climate change in a new light through research conducted in Africa. It focuses not only on shortage of resources, but also on the high price of resources and inputs, which pose a threat to security at the home level. Better natural resource management and governance, along with grassroots innovations, may increase WEF security in the area while also promoting sustainable development and lowering household vulnerabilities [72]. In Sub-Saharan Africa, researchers looked at the implications of climate change on water management, food production, and energy production. The report points out that Africa is abundant in many natural resources, including energy and mineral resources, in the context of the African situation at the turn of the century. Nonetheless, their exploitation is either completely rational or completely nonsensical and the goods are often transported outside of Africa. The continent’s deficient infrastructure, institutional and administrative capabilities, and a declining human development rate during the 1990s have all contributed to a slew of issues. Africa depends on agricultural output for both domestic consumption and export earnings. Agriculture, on the other hand, is threatened by desertification and the competitiveness of foreign markets. The region needs foreign assistance as well as development plans centered on WEF security [66]. Although the outlook is poor, Africa is nevertheless changing. Ethiopia, in Northeast Africa, is investing in agricultural modernization; biomass provides more than 90% of the country’s energy, which is produced by hydroelectric power facilities that the government is building. Water conflicts for agricultural activity and energy generation exist in the nation, as they do throughout Asia [73]. There are many issues in the vicinity of Lake Tana, Ethiopia, due to rising agricultural output and the growing need for energy for irrigation, transport, and storage. Therefore, to deal with this, we must switch to using hydroelectricity. Since the waste generated by the agriculture sector might be utilized for bioenergy production, this situation might lead to rivalry or synergy between the WEF sectors. Though both bioenergy and agriculture use a lot of water, the nation simply does not have enough of it [67]. This challenge is also faced by other African countries. Climate change impacts several African cities, including those in Bulawayo (Zimbabwe), Cape Town (South Africa), Dar es-Salam (Tanzania), and Cairo (Egypt). Water supplies for hydroelectricity generation are at risk due to these consequences. African cities are affected by water crises as well as agricultural activity in those cities. In many places the institutional capacity of local governments is inadequate, and there are budgetary restrictions and a shortage of technical expertise to address climate change, increasing the difficulties in executing the WEF nexus. The outcome is a widening of the gap between the poor and those with more financial resources, with increasing food and energy costs and restricted access to potable water [74]. South Africa follows a global trend of increasing food prices. Currently, 60% of South Africa’s households face some degree of food insecurity [75]. The price of power was up by about 24% between 2007 and 2008, hurting all economic sectors and all consumers. The Producer Price Index (PPI) indicates that gas and water costs rose by 96% and electricity costs by 177% over the course of a year. Since the cost of other food manufacturing and processing items has also climbed, food costs have gone up as well. Due to the rising energy costs, both agricultural productivity and water collection, transportation, and distribution are negatively affected. This raised the price of water by 60% as a result. In terms of food production and security, rising water costs have created more issues due to a larger shortage of water [69]. A number of new projects have emerged to address South Africa’s nexus issues. The goal of this research is to more efficiently irrigate pastures and provide nitrogen, leading to reduced fertilizer usage and more effective irrigation equipment. Conveniently, using animal manure with rainwater collecting is used to make biogas and water-soluble fertilizer, with the latter being used for household purposes [69]. Water is a critical social and environmental resource for Africa. As the population of the Nile basin grows, other resource consumption and climate change cause strain on the ecosystem. The additional stresses include internal warfare, with nations bordering the Nile all having substantial fertile riparian zones. The Nile is one of the most important river systems in Africa, and its Blue Nile tributary contributes to around 60% of the total yearly flow [76]. Allam et al., developed the WEF nexus in the Blue Nile River watershed to maximize overall benefits by harnessing water and land resources most effectively. The approach studied here has the potential to help cover up to half of the basin area with rain-fed agriculture, improving the soil in the process. The model proposed by [70] results in an annual saving of around 7.55 km^3^ of the Blue Nile River water. Of the 11 irrigation schemes that were planned in Ethiopia’s master plan, three are expected to be profitable. Water saved for hydropower generation comes at the expense of greater potential rain-fed agriculture in the basin. If nations in the region can collaborate to invest in expanding agricultural efficiency and sharing advantages and expenses, then this trade-off will provide a chance for cooperation in other areas too [70]. To go forward, these small projects need to be adopted on a larger scale to benefit the nation and the surrounding areas. Virtual water commerce, transboundary management of water resources, water-use strategies and technology are promising to contend with the WEF nexus difficulties. Due to this, the nexus method is very necessary in order to facilitate long-term strategies and strategies for Africa’s food security and long-term viability.

### 6.4. Case Study of America

Climate change and ecological processes are intertwined. North America is confronted with a number of complexity and vulnerability issues related to WEF resource management. The California Delta, for instance, has a complicated ecology with over 700 species of fish and other fauna in the western United States. Water is carried from northern to southern California in this area, which costs a lot of energy, and the Delta is used to produce food. However, problems include: (1) water distribution dependability is deteriorating; (2) increased earthquake susceptibility, as well as the danger of dikes and floods; (3) agricultural land flooding; and (4) water availability, energy generation, food production, and biodiversity are all influenced by causes such as increasing invading species and native species injury. These difficulties in the Delta might have a significant influence on California’s drinking water and agricultural food supply [77]. Mexico is dealing with a multitude of WEF resources and biodiversity protection concerns [78]. Mexico is an oil exporter with the fourth biggest shale gas reservoir in the world. It contains vast swaths of dry land, where 77% of the population lives and 87% of the country’s GDP is created. However, just 31% of the country’s rainfall is utilized, contributing to aquifer overexploitation. Furthermore, neoliberal free trade policies have permitted the importation of heavily subsidized food, as well as the rural-to-urban and global peasant movements. As a result, severe occurrences like storms and droughts, which are driven by climate change, have detrimental social and economic consequences. The development of societal instability is aided by organized crime. Water shortages, very depletable aquifers, deforested regions, natural catastrophes, high food costs, poor government institutions, high energy costs and shaky governance all contribute to increased poverty and migration in Central America’s cities, posing a danger of social instability [72]. Brazil is one of the nations in South America that might be impacted the most by climate change, and it provides an illustration of the interconnection amongst WEF systems [79]. This is because climate change has a significant correlation with environmental and social deterioration, and agriculture is the country’s primary economic sector. In terms of temperature changes, the Amazon area may see a 6° C rise, resulting in the loss of biodiversity and functional ecosystems [80]. Brazil may also see significant changes in precipitation, with a rise of roughly 60% in the country’s southern areas and a decrease of nearly 40% in the country’s northern parts. Changes in precipitation patterns in this nation may result in many water problems, including dwindling reservoir levels and, subsequently, hydroelectric power output, which accounts for around 62% of total energy output. While this energy source produces less greenhouse gas emissions, it is susceptible to environmental challenges. Changes in precipitation patterns may necessitate migration, such as the planting of crops like maize, sugar cane, and soybeans for sale or further planting somewhere else [74]. Due to the large worldwide demand for liquid biofuel made from these crops, Brazilian production has expanded dramatically over the past decade, owing to the enhanced impetus provided by regulations encouraging the development of biofuels that do not need the use of fossil fuels. This need is resulting in a significant shift in agricultural output, with an emphasis on the development of biofuels, a sector in which Brazil is a world leader. Food insecurity might develop as a result of this tendency [81,82]. The WEF nexus relationships were examined in four locations of Chile now afflicted by climatic variability and water shortages. They determined that the Antofagasta area is characterized by two distinct characteristics that influence the water–energy relationship: high copper ore yields and severe aridity. This link should be strengthened in the future since the region’s primary supply of water to manufacture copper and for human use is desalination. Copiapo is an example of a WEF nexus that is under strain as a result of intense use of water resources, especially groundwater. The main area exemplifies how the WEF nexus is impacted by intense rivalry from the urban sector, which is expected to develop further as a consequence of global warming. Water is the foundation of the WEF nexus in the Maule area, supplying supplies for agriculture and hydropower production. Thus, western South America has distinct traits and ecosystems from the rest of the continent, necessitating the implementation of special measures to reduce the effects related to the World Economic Forum Nexus. Consideration of the WEF nexus should offer a scientific basis for policy formation and should strive to integrate current activities across the three sectors. This demonstrates the critical significance of researching stochastic aspects, since changes in the water system may have an immediate effect on the food and energy systems.

## 7. Scientometric Analysis

This research includes a scientometric examination of the water, energy, and food nexus, as well as a bibliometric analysis of the overall intellectual structure of the WEF nexus. The research included all of the most popular countries as well as a wide range of keyword occurrences. For a current study on the use of WEF nexus, Scopus bibliometric data was used to compile the bibliometric data. The VOS viewer is used to achieve the goals of the current research, as shown by the findings. A new research is conducted in the VOS viewer, which is configured to generate a map utilizing data from bibliographic database files [83]. The CSV file obtained from Scopus is loaded into VOS viewer and analyzed in a few simple steps while preserving data integrity and reliability. Key words and countries mapping is shown below

### 7.1. Keyword Mapping

**Figure 7:**
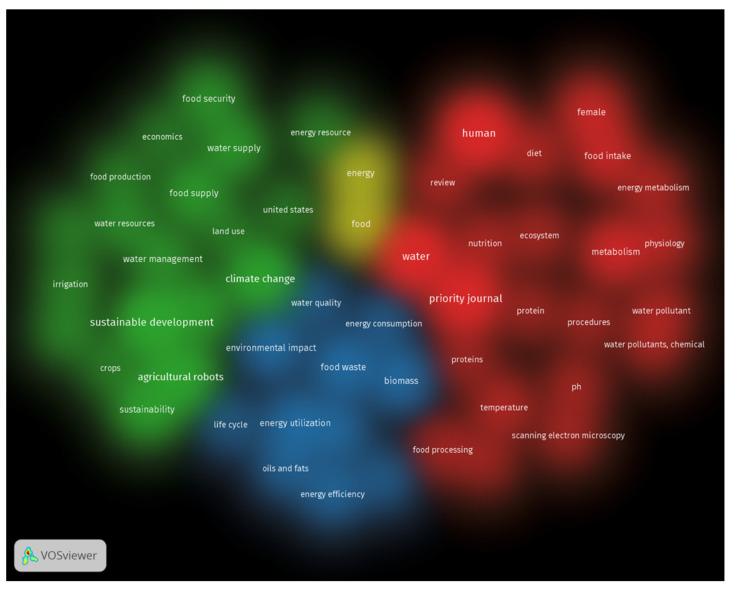
Density visualization of keywords. Green: sustainable development and water management; red: water and food intake; yellow: water and food; blue: environmental impact.

The keywords are a valuable research resource since they assist in identifying and reflecting on the subject being studied. To conduct the research, the “kind of analysis” was determined to be “co-occurrence,” and the “unit of analysis” was determined to be “all keywords.” The minimum number of times a phrase may be used was set at 50 to assure that no phrase was ever used fewer than 50 times. As a result of these restrictions, it was found that only 72 words out of 19,146 met the threshold for inclusion. According to the researchers’ data, the phrases water, climate change, food, and sustainable development are the ones that occur the most often. Figure 7 displays the co-occurrence of keywords in a density visualization, their connections with one another, and the density of their correlation frequency density in the network. Although the location of the keyword node in Figure 7 indicates how often a word appears in published works, its size indicates how often it appears in those works. Aside from that, the visualization shows that the aforementioned keywords have bigger nodes than the others, indicating that they are the most relevant terms in the study of the water–energy–food nexus. It has been decided that many keywords should be visually distinguishable in the network to indicate their co-occurrence in a variety of publications. There was a total of four clusters identified, with each group represented by the colors: green indicates sustainable development and water management; red presents water and food intake; yellow shows water and food; blue depicts environmental impact.

### 7.2. Countries Mapping

Certain nations have contributed more to modern research than others have and continue to do. The network of visualizations was built to aid readers in visualizing locations dedicated to environmentally responsible construction. The method of analysis was bibliographic coupling, and the unit of analysis was countries.

**Figure 8:**
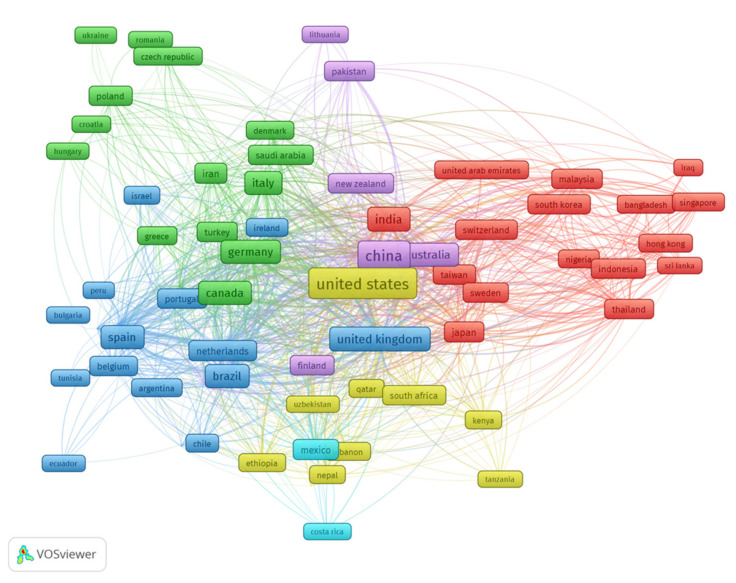
Network visualization of countries.

A nation’s minimum need for papers was set at five, and 71 of the 124 countries met this criterion. The United States, China, and the United Kingdom provided the most papers overall, with 395, 380, and 159 papers, respectively. The United States, China, the United Kingdom, and Australia received the most citations, with 611, 694, 365, and 255 citations, respectively. The number of publications, citations, and overall link strength all indicate a country’s influence on the growth of the current research area. The total strength of the relationship indicates the extent to which the papers of one country influenced the papers of the other countries covered in these studies. As a consequence, the aforementioned nations were considered to have the highest influence on drought monitoring. Figure 8 illustrate the countries’ interconnectivity and density of countries connected by citations. The frame’s size indicates a country’s contribution to the field. Additionally, the density graph demonstrates that the countries with the most participation had a higher density. The graphical representation of participating countries will assist future academics in establishing scientific collaborations, developing joint venture reports, and sharing novel approaches.

## 8. Discussion

This article analyzed water–energy nexus techniques and divided them according to their nexus scopes into four broad categories. These methods are determined by their primary objectives. As seen in Table 2, research on the water–energy–food nexus has grown in size and breadth over the last decade, with an increase in both the number of studies and the scientific community’s capability to examine water and energy interconnections effectively at a greater resolution. This may be observed on a micro level, such as in the use of water and energy in industry and infrastructure, to a macro level, where evaluations are used to support regional resource management policies. Environmental effects have been an increasingly prevalent and emphasized consequence of these in-depth case studies. This is a significant advance in terms of resource management benchmarking. This review contains various instructive instances that may aid in the advancement of research on WEN dynamics. The studies used stakeholder theory principles to build a systematic technique for quantifying water and energy links and identifying important players and groups capable of bridging inter-organizational networks for water and energy planning. PRIMA encompasses aspects that aid in understanding, governing, and implementing the nexus. A major role of an integrated model will be long-term scenario analysis. Reasonable scenarios are always built on a long-term examination of previous water and energy data. At the level of controlling the nexus, CGE and ZeroNet DSS are integrated. The data intensities necessary to employ the WATER, TRBNA, and PRIMA, on the other hand, are all rather large. The WEN and ZeroNet DSS are regarded to be the most appropriate available approaches for the WEN, with each focusing only on the water system and energy sectors. Table 3 highlights the primary WEF nexus recommendations based on the scientific literature evaluated in this research. These proposals are organized into three categories: public policies, research and development, and practice. Those relating to policy, on the other hand, should be executed by the government, based on scientific facts, and backed up by the participation of diverse stakeholders. The research and development components are critical in supporting governance structures and practices aimed at achieving sustainable development and mitigating climate change, as well as public policy and practices. For the upkeep of the WEF systems, public policies give recommendations and suggest which acts should be promoted or forbidden. Finally, the practice component is concerned with strategic manufacturing measures that correspond to WEF nexus sustainability requirements. It is also in charge of distributing innovations in the marketplace, enabling the spread of innovations, information, and goods that will help society transition to a more sustainable state. To build long-term policies and strategies that help support food security and sustainability for Asia, Africa, America, and Europe, the nexus should be taken into account since it provides a scientific foundation for policy development and aims to integrate existing efforts across the three sectors. Changes in the water system may have an instantaneous impact on the food and energy systems, demonstrating the essential need of studying stochastic features. To offer a scientific foundation for policy formulation and to integrate current activities across the three sectors, it is recommended that the WEF nexus be examined. Stochastic concerns related to the water system must be studied due to its connection to the energy and food systems. Scientometric analysis was used to determine the pertinent research topics, the countries mapping, and the co-occurrence of keywords, in the water–energy–food nexus. The top five most often used terms in the literature were water, climate change, food, and sustainable development. The visualization network clearly demonstrates the importance of water, energy, and food in relation to climate change, with the United States, China, and the United Kingdom providing the most papers.

## 9. Conclusions

The four kinds of nexuses discovered were all linked to water, with the majority of them focusing on fresh water, such as river water, rainwater, reservoir water, groundwater, and seawater and most of them relating to terrestrial activities such as agriculture production and wastewater treatment. PRIMA encompasses aspects that aid in understanding, governing, and implementing the nexus. A vital role for an integrated model will be long-term scenario analysis. Reasonable possibilities are always based on a long-term review of previous water and energy data. The Integrated CGE and ZeroNet DSS are all in charge of controlling the nexus. However, the data intensities necessary to employ the WATER, TRBNA, and PRIMA are all substantial. WEN and ZeroNet DSS are regarded as the most appropriate accessible approaches for the WEN and ZeroNet DSS, with each concentrating only on the water system and energy sectors, respectively. While numerous studies seek to establish new methodologies and frameworks for fully assessing interactions between water, energy, and other components, none offers a unified framework for conducting nexus research. According to research and reports, methodologies presented in this review are in the understanding stage, with a focus on the quantitative analysis of the WEN. However, this is not the case for many areas and nations. The problems will be the most significant issue in controlling or implementing the WEN. The extent of the nexus components being analyzed, according to the nexus studies in this study, continues to increase, spanning from water, energy and food, land, nutrients, climate, and more. This improvement improves the resolution and analytical capability of resource and environmental interactions, but it also raises data consumption and model complexity. Given the vast number of analytical tools, frameworks, and methodologies for applied WEN work, as well as the vast number of prospective applications, there is an obvious need to increase our capacity to identify and analyze the abilities, strengths, and shortcomings of current techniques. Scientometric analysis show that China and the United States are the countries with highest number of research articles and citations in the field of water–energy–food nexus. Further this review also studies the WEF for America, Asia, Africa and Europe. Increased global demand for natural resources, along with climate change, has posed significant challenges to European urban growth and sustainability. It is required to adopt a transboundary approach to address the WEF nexus problems and promote sustainable development. The researchers studied the effects of climate change on water management, food production, and energy generation in Sub-Saharan Africa. In the United States, water is transported from northern to southern California at a high expense of energy, and the Delta is utilized to grow food. The strategy described in this research aims to fill a knowledge gap. Future suggestions are provided below.

- Additional research in this area might help a broader range of stakeholders better use current information to enhance their water and energy resource management.
- It might also assist the scientific community to better identify, correct, and prevent scientific errors in their methods and provide better evidence for decision making, management, and policy alternatives. This might pave the way for future research to place a greater emphasis on controlling and executing the nexus.
- To grow, efforts should be undertaken to both increase and broaden the research centered on what this paper refers to as historically predominant paradigms, as well as to seek out new and more holistic approaches to addressing linkages between water, food, environmental security and energy concerns.
- Some common criteria should be followed to prevent mismatches, reduce disparities, and compare the abilities, strengths, and shortcomings of current techniques. A further in-depth investigation focused on the nexus modeling, identifying their strengths and weaknesses, is strongly advised.
- Furthermore, to persuade policymakers to adopt a WEF nexus thinking and strategy, management scenarios should be provided from an economic, environmental, i.e., greenhouse gas emissions, and social viewpoint, in addition to resource usage efficiency.
- To better manage natural resources for the benefit of humanity, which is impossible without considering the social context, a better understanding of the three critical pillars of numerical modeling, stakeholders, and technology is required.

## Figures and Tables

**Table 1:** Challenges and solutions of water–energy–food nexus.

Challenges	Solutions
Inefficacy of IT platforms	Use domain-spanning software
Lack of system-wide policy and law	Developing a model of agricultural complex and industrial appropriate allocation, as well as integrated cost in the energy and water sectors.
Subsystems with a large amount of data	Implementing data-mining algorithms
Inadequate standards and legislation	Creating sub-specialty committees to solve the gap
Uncertainty in data	Using uncertainty modeling techniques like Scenario generation and stochastic programming.

**Table 2:** Different models for water–energy–food nexus.

Method	Type of Model	Software	Objective	Nexus Degree of Difficulty
System dynamic approach [84]	Integrated model	No software	Regional water and energy resource management for the long term.	Understanding
Jordan’s framework [85]	Integrated model	No software	In Jordan, link decision-making to increased water and energy efficiency.	Governing
RRP [86]	Quantitative analysis model	No software	Water flow and temperature effects on the power system.	Understanding
WEAP-LEAP	Integrated model	No software	Water and energy demand are impacted by policy.	Understanding
SPATNEX-WE [87]	Integrated model	No Software	Document the movement of energy and water, as well as the consumption of water and energy, through the system.	Understanding
MA [88]	Quantitative analysis method	No software	Calculate the amount of materials and energy that go through our nation’s electrical, water, and wastewater networks.	Understanding
Nexus Assessment 1.0	Quantitative analysis model	Online tool	Examining the relationship between qualitative and quantitative.	Governing
ZeroNet DSS [55]	Integrated model	Several freesoftwares	Tool for managing resources in the basin	Governing
Modified SWAT [89]	Integrated model	Open-source model	Water provision in transboundary areas in each sector of the economy.	Understanding
PRIMA	Integrated model	Velo	Analyze how climate, energy, water, and land interact to make decisions.	Implementing
TRBNA	Integrated model	UNECE; NS	Consider trans-boundary river basins to further assess the WEFEN.	Implementing
CLEWS	Integrated model	KTH; Open-source tool OSeMOSYS	Assess climate effects on natural resources and give support for resource-related policy	Implementing
GCAM-USA [90]	Integrated model	Open-source tool	More extensive long-term examination of water withdrawals and water demands in the US states’ power sectors has been done.	Governing

**Table 3:** Key concepts of review.

Authors	Purpose	Recommendations
Lal [91]	Reduces waste by increasing WEF resource efficiency.	By using effective harvesting, transport, and refrigeration methods, you may reduce post-harvest losses.
Hang et al. [92]	Minimize waste, boost water securityas well as provide clean water to every single.	The technology may also be used to improve water usage efficiency, such as by using irrigation techniques that focus on water conservation, including hydroponics, micro irrigation. Rainwater storage is also helpful.
Kurian [93]	Make stakeholders aware of the climate change effects on WEF’s security, by passing on relevant information as well as capacity development.	Use the holistic/multi-sectoral and multi-disciplinary approach to holistic capacity development nexus from the WEF.
Bayley et al. [94]	Reduce competition for urban land through optimizing usage of urban areas. increases the Earth’s Resilience to the WEF nexus, as well as the complexity of that domain.	Boost the local fresh food supply, reduce energy use and improve the efficiency of packaging transportation.
Conway et al. [95]	Efforts to boost multidisciplinary and multisectoral research, including decisions about policy, by providing access to data and information on the Nexus components.	Fosters policies and managerial structures that enable WEF links and integration.
Berchin et al. [96]	Migration is a human right, and supporting immigration policies are vital for the present environment. The present changes in the environment, including the WEF nexus, pose significant challenges for the poor, who are most in need of help.	Help human migration due to environmental disturbances and climate change by implementing plans and regulations.
Rasul [97]	Using the model findings to assist policy makers with new methods for tackling the Nexus	Resilience, resource efficiency, and sustainability may all be improved by connecting various sectors and scales.
Ringler et al. [98]	Water-saving practices, including the sustainable management of water resources, have the potential to decrease waste, provide water security, and make water available to everyone.	Conservation agriculture, micro irrigation, and rainwater storage may all be used to enhance the efficiency of water usage.
Hussien et al. [99]	By creating an organization that allows communities of practice to develop around the WEF nexus, it is possible to get information on the community’s local and regional concerns, as well as providing multisector, interdisciplinary, and multistakeholder solutions.	Promote community practice of multi-stakeholder participation inside the WEF nexus.
Smajgl et al. [28]	Migration is a human right, and supporting immigration policies are vital for the present environment. The present changes in the environment, including the WEF nexus, pose significant challenges for the poor, who are most in need of help.	Help human migration due to environmental disturbances and climate change by implementing plans and regulations.
Pittock et al. [100]	It may help managers make long-term decisions about sustainable development by using a scientific model of the WEF nexus.	Design an integrated data-based model for the WEF nexus, where this will provide the necessary information to assist with decision making and forecasting.
